# Prognostic Significance of Neutrophil to Lymphocyte Ratio in Oncologic Outcomes of Cholangiocarcinoma: A Meta-analysis

**DOI:** 10.1038/srep33789

**Published:** 2016-10-03

**Authors:** De-Wen Tan, Yan Fu, Qi Su, Ming-Jun Guan, Po Kong, Sheng-Qiang Wang, He-Ling Wang

**Affiliations:** 1 Department of General Surgery, Shengjing Hospital Affiliated with China Medical University, Shenyang, Liaoning 110004, China; 2 Department of Head and Neck Oncology, Sun Yat-sen University Cancer Center, State Key Laboratory of Oncology in South China, Collaborative Innovation Center for Cancer Medicine, Guangzhou, China

## Abstract

Increasing evidence indicates that the neutrophil to lymphocyte ratio (NLR) is a useful biomarker of long-term outcomes in patients with cholangiocarcinoma. However, the prognostic role of NLR in patients with cholangiocarcinoma remains unclear. Thus, the current meta-analysis was undertaken to clarify the correlation between NLR and overall survival (OS) in cholangiocarcinoma, and a comprehensive literature research was conducted to understand the association of NLR and prognosis of cholangiocarcinoma. The hazard ratio (HR) with 95% confidence interval (CI) was used to assess OS. The synthesized HR of 1.449 (95% CI: 1.296–1.619, *P* < 0.001) indicated that a high NLR had an unfavourable effect on OS. Overall, this meta-analysis suggested that elevated preoperative NLR is associated with poorer rates of survival in cholangiocarcinoma patients.

The incidence and mortality rates of the malignant tumour cholangiocarcinoma (CCA) are increasing worldwide^1^,^2^, with approximately 5000 CCA-related deaths occurring per year^3^. Although patients undergo curative-intent surgery or adjuvant therapies including systemic chemotherapy and radiotherapy for palliation of CCA, their clinical outcomes remain poor^4^. Several trials have shown that clinicopathological factors including tumour size^5^,^6^, intrahepatic satellite lesions^6^,^7^, lymph node metastasis^8^, vascular invasion^6^ and resection margin involvement are associated with poor survival^9^. However, the available data are largely derived from retrospective observational studies mostly from small, single-institution series, so they are not applicable to clinical practice and cannot be validated externally. Recently published evidence suggests that systemic inflammation is related to poor survival in patients with various types of malignancies^10^,^11^,^12^. The neutrophil to lymphocyte ratio (NLR) is one of the inflammatory parameters that has been reported to be of prognostic value for some solid tumours, including CCA^13^,^14^. However, because of variation in study design and limited sample sizes, studies have yielded conflicting results on the use of NLR to predict OS in patients with CCA^13^,^15^. Thus, a meta-analysis to estimate the prognostic value of NLR in these patient groups is of significance.

## Results

### Selection and characteristics of included studies

**Figure 1:**
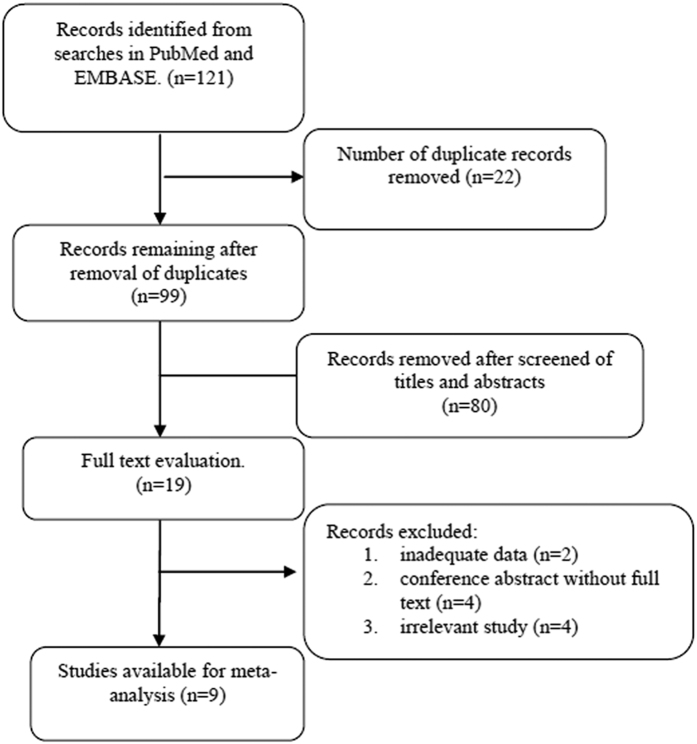
Flow chart of literature search and selection.

The flowchart of the study selection process is shown in Fig. 1. A total of 121 records were identified from an initial comprehensive literature research. Then, 99 of these 121 articles were included after removing duplicates. Eighty studies were excluded after their titles and abstracts were screened. After full text assessment, 2 more studies were excluded because they did not provide adequate data for calculating the HR and 95% CI, and 8 studies were excluded because they were either conference abstracts or not relevant. Thus, 9 studies that met our selection criteria with a total of 2093 patients with CCA^13^,^16^,^17^,^18^,^19^,^20^,^21^,^22^,^23^ were finally included in this meta-analysis. Among the 9 articles included, 1 article by Hamed *et al.* could be divided into two “sub-groups,” given that it provided survival data related to NLR and OS categorized as ampullary carcinoma and cholangiocarcinoma depending on the anatomic location of the tumour; thus, we designated them as Hamed1 and Hamed2. Similarly, 1 article by McNamara *et al.* could be segmented into three “sub-groups” including three cohorts and reported the HR and 95% CI; we designated them McNamara1, McNamara2 and McNamara3. The main characteristics of the 9 articles, which included 12 studies, are summarized in Table 1. Seven studies came from Western countries, including the United Kingdom, Canada and Romania. The remaining 5 studies were from Japan, Korea, and China. NLR was recorded from the pre-treatment data in all studies. HRs and 95% CIs had been obtained via multivariate analysis in 7 studies and were recorded from the original literature. The HRs for the 5 remaining studies were deduced from survival curves or other data. The scores of study quality estimated using the Newcastle Ottawa Scale (NOS) for quality assessment ranged from 5 to 7.

### Meta-analysis results

**Figure 2:**
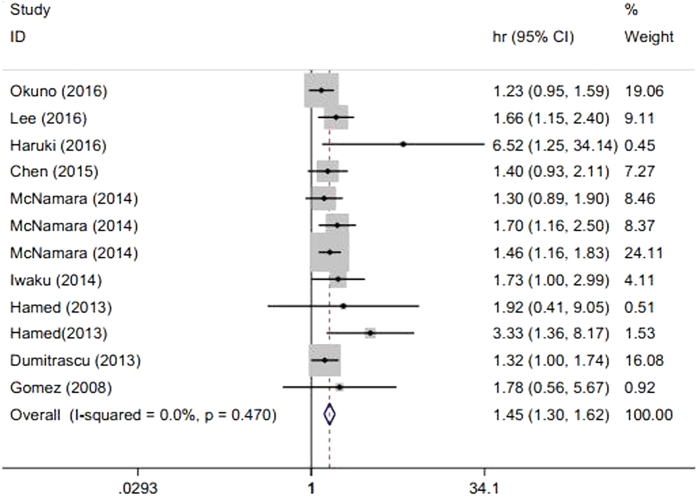
Meta-analysis of the association between elevated NLR and OS in patients with CCA.

No obvious between-study heterogeneity was detected (I^2^ = 0.0%, *P* = 0.47), and thus, a fixed-effects model was applied to estimate pooled HR. The combined HR of 1.449 (95% CI: 1.296–1.619, *P* < 0.001) suggested that patients with elevated NLR tended to have poor OS. The forest plot for this analysis is shown in Fig. 2.

Subgroup analysis was performed by therapeutic intervention (surgical and mixed), and the pooled estimates displayed that elevated pre-treatment NLRs predicted poor prognosis for patients both in Western countries (HR = 1.360, 95% CI: 1.112–1.609) and Eastern countries (HR = 1.421, 95% CI: 1.211–1.631). Further, high NLR predicted a poor prognosis in patients treated with both surgical (HR = 1.353, 95% CI: 1.099–1.607) and mixed (surgical and non-surgical) interventions (HR = 1.424, 95% CI: 1.217–1.691). On performing subgroup analyses stratified by cut-off value, we found that increased NLR was a negative predictor for patients with cut-off values ≥4 (HR = 1.724, 95% CI: 1.215–2.233) and cut-off value <4 (HR = 1.360, 95% CI: 1.191–1.529). Subgroup analysis by the NOS score of the studies showed that a high NLR indicated poorer OS in CCA patients for studies with both NOS score ≥7 (HR = 1.396, 95% CI: 1.235–1.556) and NOS score < 7 (HR = 1.311, 95% CI: 1.078–1.544). Finally, stratification by sample size showed that the combined HR was 1.394 (95% CI: 1.212–1.576) for studies with more than 200 cases and 1.402 (95% CI: 1.062–1.743) for those with less than 200 cases (Table 2).

### Publication bias

**Figure 3:**
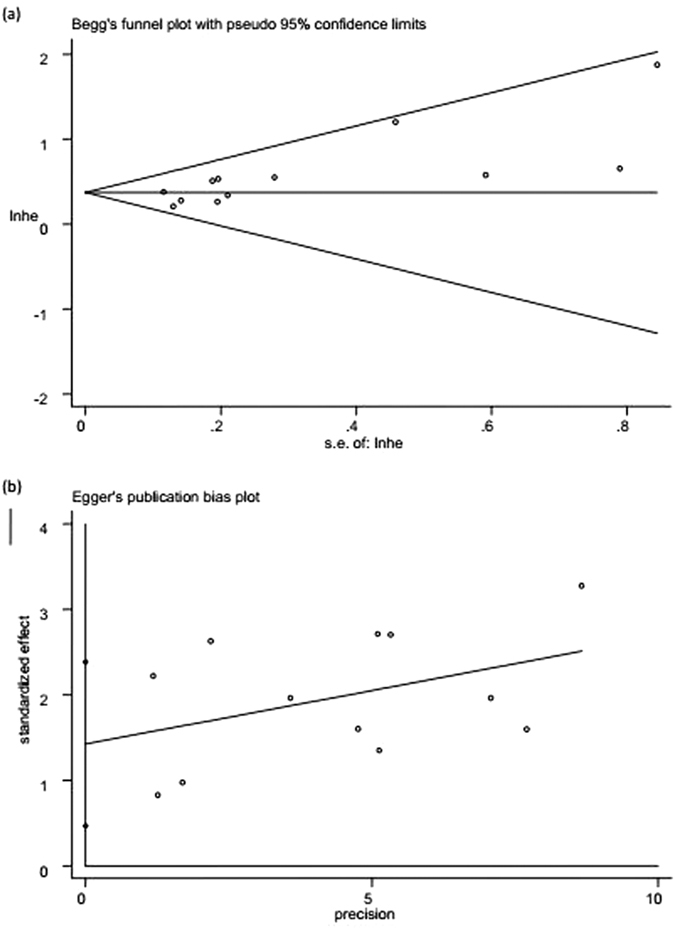
Begg’s (**a**) and Egger’s (**b**) funnel plot for assessing potential publication bias.

**Figure 4:**
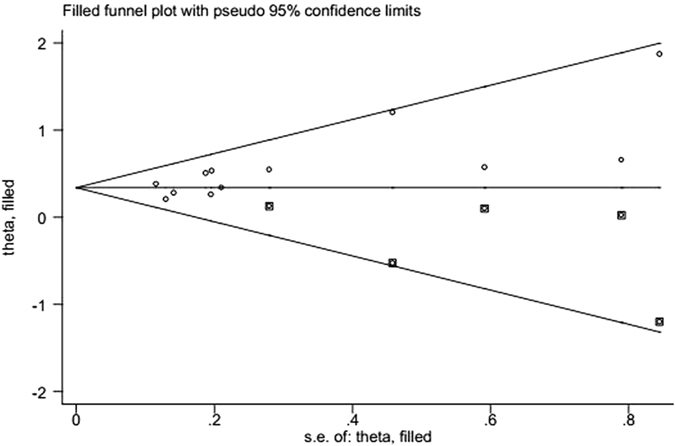
Funnel plot adjusted using the trim and fill method for OS. Diamonds: included studies; diamonds in squares: presumed missing studies.

Begg’s funnel plot and Egger’s test linear regression test suggested the visual assessment of overt publication bias had statistical significance for the included studies (P > |t| = 0.008; Fig. 3). Therefore, we further performed a “trim and fill” analysis and found that filling 4 unpublished studies did not significantly change the recalculated combined HRs of OS (HR = 1.402, 95% CI: 1.260–1.560; *P* < 0.001; Fig. 4).

## Discussion

The meta-analysis conducted in the present study on 12 studies with a total of 2093 patients with CCA demonstrated that a high NLR is associated with significantly poor OS. Similar to our study, 2 recent meta-analyses confirmed the prognostic value of NLR for pancreatic cancer and non-small cell lung cancer^24^,^25^. To our knowledge, ours is the first meta-analysis assessing the prognostic role of NLR in CCA.

Inflammation plays an important role in tumour growth, including matrix degradation and cancer progression and causes immunosuppression and enhances angiogenesis^26^. This microenvironment potentiates and enhances the neoplastic risk and ultimately promotes metastatic spread^12^. Neutrophils and immunocytes related to inflammation mediate communication between the microenvironment and tumour cells. Different categories of cells play distinct roles in the systemic inflammation response. Studies have shown that neutrophils promote the survival and proliferation of cancer cells by secreting many inflammation mediators such as tumour necrosis factor, interleukin 1, interleukin 6 and vascular endothelial growth factor^27^,^28^,^29^. However, lymphopenia is vital in the immune defence against tumour cells^30^. The infiltration of CD4+ T cells triggers the immune activation of CD8+ T cells^31^, and activated CD8+ T cells cause apoptosis of cancer cells by releasing cytotoxic factors^32^. These findings collectively indicate that it is reasonable to assume that neutrophilia and lymphocytopenia are a potential indicator of prognosis for estimating the systemic inflammatory response and outcome of individual patients. As an indicator of the balance between tumour destruction and tumour protection, NLR is a significant prognostic factor.

Although we comprehensively evaluated the association between the NLR and CCA, this meta-analysis has some limitations. First, a publication bias obviously exists since small-scale studies are prone to remain unpublished and could account for language limitations in the inclusion criterion or selective publication. Second, a selection bias is impossible to avoid because most of the studies included in this meta-analysis were retrospective. Third, the included articles use different NLR cutoff levels and determine these levels using various methods. Therefore, the threshold value of NLR should be standardised in future trials and in clinical practice. Fourth, the data were not adequate for us to examine the relationship between NLR and the clinicopathological parameters of the tumour. Finally, the HRs and Cis had to be deduced from survival curves in 6 studies because these studies did not report these parameters directly.

In conclusion, our study demonstrated the importance of NLR as a predictor of OS in patients with CCA. The NLR is easily determine from the widely available findings of routine blood tests, so it can be extensively used as a novel predictive factor for cholangiocarcinoma. Further large-scale research and standardised investigations are warranted to confirm our findings.

## Methods

### Literature research

We performed a search of articles published in PubMed and EMBASE up to March 2, 2016. The relevant studies were identified using the following search terms:

1. “neutrophil to lymphocyte ratio,” “neutrophil lymphocyte ratio,” “neutrophil-to-lymphocyte ratio”;
2. Bile duct OR cholangio* OR biliary tract OR Klatskin OR “ampulla of Vater”;
3. Cancer OR adenocarcinoma OR carcinoma;
4. #1 AND #2 AND #3.

### Study selection criteria

The entire selection process was performed independently by two authors (T.D.W. and F.Y.), and a third author (S.Q.) was consulted to resolve any discrepancies. We included studies that met the following selection criteria: (1) investigation of the prognostic value of NLR in CCA; (2) data available for calculating survival estimates, such as HR with 95% CIs, or *P* values and other data that could be used to calculate these values^33^; (3) and availability of full text. Abstracts, meetings or case reports were excluded.

### Data extraction and quality assessment

Data extraction and quality assessment were conducted independently by two authors (T.D.W. and G.M.J.). Any disagreement was resolved by discussion and consensus. The investigators extracted the following data from the 12 studies: names of the first authors, publication year, sample sizes, participant characteristics, and endpoints with their corresponding HRs and 95% CIs. The NOS was used to evaluate study quality.

### Data synthesis and analysis

HRs and 95% CIs from each study were used to calculate pooled HRs. Cochran’s Q test and Higgins’ I-squared statistics were used to test the heterogeneity of the combined HRs. If heterogeneity was observed, the random effects model (Der Simonian and Laird method) was applied for analysis; otherwise, the HRs were pooled using a fixed-effects model. We tried to contact the authors of published studies and failed to do so for subgroup analysis, and subgroup analysis and meta-regression analyses were performed to detect and explain the heterogeneity among the results of various studies. Sensitivity analyses were performed to confirm the robustness of the study. Egger’s linear regression test and Begg’s funnel plot test were used to evaluate publication bias^34^. The trim and fill method was applied to estimate asymmetry in the funnel plot^35^. Statistical significance was set at 0.05. All statistical analyses were performed using STATA version 12.0 (StataCorp, College Station, TX, USA).

## Additional Information

**How to cite this article**: Tan, D.-W. *et al.* Prognostic Significance of Neutrophil to Lymphocyte Ratio in Oncologic Outcomes of Cholangiocarcinoma: a Meta-analysis. *Sci. Rep.* **6**, 33789; doi: 10.1038/srep33789 (2016).

## Figures and Tables

**Table 1:** Patients’ clinicopathological characteristics.

Study	Year	Area	Sample size	Survival analysis	HR (95% CI)	Treatment	Cut-off value	Summary results	NOS score
Okuno^16^	2016	Japan	534	OS	E	Surgery	3/3–5/5	Negative	6
Lee^13^	2016	Korea	221	OS	M	Surgery	5.0	Positive	7
Haruki^17^	2016	Japan	37	OS	M	Surgery	3.0	Positive	6
Chen^18^	2015	China	322	OS	M	Surgery	2.49	Positive	7
McNamara^19^	2014	Canada	179	OS	M	Surgery & Non-surgery	3.0	Positive	7
McNamara^19^	2014	Canada	161	OS	M	Surgery & Non-surgery	3.0	Positive	7
McNamara^19^	2014	Canada	220	OS	M	Surgery & Non-surgery	3.0	Positive	7
Iwaku^20^	2014	Japan	52	OS	E	Surgery & Non-surgery	4.0	Negative	5
Hamed^21^	2013	UK	74	OS	E	Surgery	5.0	Negative	5
Hamed^21^	2013	UK	69	OS	E	Surgery	5.0	Positive	6
Dumitrascu^22^	2013	Romania	197	OS	E	Surgery & Non-surgery	3.3	Negative	6
Gomez^23^	2008	UK	27	OS	M	Surgery	5.0	Positive	5

OS: overall survival; HR: hazard ratio, obtained by estimating (E); M indicates that the HR comes from multivariate analysis; NR: not reported; NOS: Newcastle Ottawa Scale.

**Table 2:** Summary of meta-analysis results.

Analysis	NO.	Model	HR (95% CI)	Ph
Overall survival	9	Fixed	1.449 (1.296–1.619)	0.470
Subgroup1: Area				
Eastern	5	Fixed	1.360 (1.112–1.609)	0.654
Western	4	Fixed	1.421 (1.211–1.631)	0.862
Subgroup2: treatment				
Surgery	6	Fixed	1.353 (1.099–1.607)	0.762
Surgery& non-surgery	3	Fixed	1.424 (1.217–1.691)	0.924
Subgroup3: cut-off				
≥4	4	Fixed	1.724 (1.215–2.233)	0.924
<4	5	Fixed	1.360 (1.191–1.529)	0.873
Supgroup4: NOS score				
≥7	3	Fixed	1.396 (1.235–1.556)	0.924
<7	6	Fixed	1.311 (1.078–1.544)	0.825
Supgroup4: Sample size				
≥200	4	Fixed	1.394 (1.212–1.576)	0.733
<200	5	Fixed	1.402 (1.062–1.743)	0.924

Ph: *P* value of the Q test for heterogeneity; No.: number of studies; HR: hazard ratio.
